# Einfluss sozialer Beziehungen auf Corona-Sorgen bei der Pflegeheim-Bevölkerung: Quantitative Querschnittsbefragung

**DOI:** 10.1007/s00391-022-02116-5

**Published:** 2022-09-28

**Authors:** Rebekka Rohner, Vera Gallistl, Vera Hartmann, Theresa Heidinger, Gerhard Paulinger, Franz Kolland

**Affiliations:** Kompetenzzentrum Gerontologie und Gesundheitsforschung, Karl Landsteiner Privatuniversität für Gesundheitswissenschaften, Dr.-Karl-Dorrek-Str. 30, 3500 Krems an der Donau, Österreich

**Keywords:** Langzeitpflege, Einsamkeit, Psychische Resilienz, Coping-Strategien, Sozioemotionale Selektionstheorie, Long-term care, Loneliness, Resilience, mental, Coping strategies, Socioemotional selection theory

## Abstract

**Hintergrund:**

Während der durch die „coronavirus disease 2019“ (COVID-19) ausgelösten Pandemie im Frühling und im Sommer 2020 stiegen die Sorgen und Ängste von Bewohner*innen der stationären Langzeitpflege vor einer Erkrankung und den Auswirkungen des Virus (Corona-Sorgen). Im Umgang mit Sorgen stellt der Kontakt zu nahestehenden Personen eine wichtige Ressource dar; dieser wurde allerdings gerade in Pflegeeinrichtungen streng reglementiert. Deshalb wird von erhöhten psychischen Belastungen ausgegangen, es mangelt jedoch an repräsentativen Daten.

**Ziele der Arbeit:**

Die Arbeit verfolgt die Fragestellung: Wie beeinflussen die sozialen Beziehungen von Pflegeheimbewohner*innen ihre Corona-Sorgen?

**Material und Methoden:**

Im Sommer 2020 wurden in ganz Österreich 259 Pflegeheimbewohner*innen eines Trägers in einer repräsentativen, standardisierten Face-to-face-Befragung interviewt.

**Ergebnisse:**

Erstens zeigen die Daten hohe emotionale Belastungen bei den Pflegeheimbewohner*innen, allerdings auch eine gewisse psychische Resilienz. Zweitens besteht ein Zusammenhang zwischen emotionaler Einsamkeit und höheren Corona-Sorgen („odds ratio“ [OR] = 2,30; *p* < 0,01). Drittens hängt auch ein häufiger telefonischer und/oder persönlicher Kontakt zu Angehörigen mit höheren Corona-Sorgen zusammen (OR = 1,32; *p* < 0,05).

**Schlussfolgerungen:**

In den aktuellen Zeiten, in denen eine Krise die nächste ablöst, braucht es mehr Wissen über die psychischen Resilienzen von Pflegeheimbewohner*innen und wie diese gefördert werden können. Außerdem braucht es mehr Wissen über die Rolle der Angehörigen, und ob diese eher ein Ausgangspunkt von Sorgen sind oder eine Umgangsstrategie mit Sorgen darstellen.

## Abstract (en)

**Background:**

During the pandemic caused by the coronavirus disease 2019 (COVID-19) in spring and summer 2020, the anxiety and concerns of residents living in long-term care facilities about contracting and falling ill from the virus (corona concerns) increased. Contact to close persons represents an important asset in coping with concerns or anxiety; however, this was strictly regulated particularly in nursing facilities. Therefore, an elevated psychological burden was assumed; however, there is a lack of representative data.

**Objective:**

This study aims to answer the question: how do social contacts influence the corona concerns of long-term nursing home residents?

**Material and methods:**

In summer 2020 a representative sample of 259 long-term nursing home residents in the whole of Austria participated in a standardized face-to-face interview.

**Results:**

Firstly, the data show a high emotional burden in nursing home residents; however, many residents also demonstrated a certain degree of mental resilience. Secondly, there was an association between emotional loneliness and higher corona concerns (odds ratio, OR = 2.30; *p* < 0.01). Thirdly, a frequent contact with relatives via telephone or in person also related to higher corona concerns (OR = 1.32; *p* < 0.05).

**Conclusion:**

In the current times, when one crisis is replaced by the next, more knowledge is needed about the mental resilience of long-term nursing home residents and how it can be promoted. Furthermore, more knowledge is needed about the role of relatives and whether they are more the origin of concerns or they represent a strategy for coping with concerns.

## Einleitung

Die durch die „coronavirus disease 2019“ (COVID-19) ausgelöste Pandemie hat sich in den letzten Jahren zu einem besonderen Thema der gesundheitsbezogenen Sorgen im Alter („health anxiety“) entwickelt. Unter gesundheitsbezogenen Sorgen werden gemeinhin Befürchtungen über eine Verschlechterung der Gesundheit und das potenzielle Vorhandensein von Krankheiten bezeichnet. Diese allgemeinen Sorgen können Ausprägungen von schwachen gesundheitsbezogenen Bedenken bis hin zu hypochondrischen Krankheitsüberzeugungen aufweisen [21]. Während sich Erstere durchaus auch positiv auf das Gesundheitsverhalten auswirken können, stellen Letztere eine psychische Erkrankung dar [23]. Zu Beginn der Pandemie wurden ältere Menschen und v. a. Pflegeheimbewohner*innen als Risikogruppe für einen schweren Verlauf der Erkrankung adressiert [12], was mit strengen Einschränkungen der sozialen Kontakte einherging sowie mit erhöhten psychischen Belastungen und Ängsten in Verbindung gebracht wurde [5].

Im Rahmen bisheriger Forschungsprojekte wurden die Bewohner*innen von stationären Langezeitpflegeeinrichtungen vergleichsweise selten direkt zu ihren Corona-Sorgen befragt. Die vorhandenen Studien weisen v. a. ein qualitatives Design auf, und ihre Ergebnisse zeigen, dass die pandemiebedingten Einschränkungen bei Pflegeheimbewohner*innen zur Verringerung der Lebensqualität sowie zur Verstärkung von Depression und Angst geführt haben [14]. Bewohner*innen berichten von Angst und Einsamkeit, die v. a. durch fehlende soziale Kontakte zu nahestehenden Personen und fehlende soziale Aktivitäten verstärkt wird [19]. In der Publikation einer weiteren Studie wird beschrieben, dass sich die sozialen Beziehungen durch die Einschränkungen deutlich gewandelt haben sowie neue Unsicherheiten und Ängste erlebt wurden [8].

Ergebnisse von Studien mit der älteren Bevölkerung in Privathaushalten unterstreichen diese Befunde und zeigen, dass ältere Menschen, die Einsamkeit erlebt hatten, signifikant höhere Corona-Sorgen aufweisen als jene, die keine Einsamkeit angaben [18]. Ebenfalls konnte in einer Langzeitstudie von 2015 bis 2020 festgestellt werden, dass die Pandemie v. a. bei einsamen älteren Menschen zu höheren allgemeinen Ängsten geführt hat [9]. Resultate aus Studien mit älteren Menschen in Privathaushalten unterstreichen die Bedeutung von sozialen Beziehungen als protektiven Faktor gegenüber Sorgen, die mit der COVID-19-Pandemie zusammenhängen.

Erklärt werden können solche Befunde mit Referenz auf die sozioemotionale Selektivitätstheorie [7], die davon ausgeht, dass soziale Beziehungen eine protektive Wirkung haben, da sie zur Regulation von negativen Emotionen beitragen. Die Theorie geht davon aus, dass gerade bei Menschen im höheren Alter enger Kontakt zu nahestehenden Menschen wichtig für das Abfangen von negativen Emotionen, Ängsten und Sorgen ist. Zentrale Annahme der Theorie ist, dass die Motivation für Handlungen entweder auf die Aneignung von Wissen oder die Regulation von Emotionen ausgerichtet ist. Ältere Personen nehmen die verbliebene Lebenszeit als begrenzt wahr und fokussieren emotionale Ziele. Carstensen et al. konnten [7] in mehreren Studien deutlich machen, dass enge, jahrelange Beziehungen bei Menschen im höheren Alter von großer Bedeutung für das Erleben von sozialer Einbindung und positiven Emotionen sowie den Umgang mit negativen Emotionen sind.

Gerade bei Bewohner*innen von Pflegeheimen unterlagen die sozialen Kontakte jedoch strengen Einschränkungen. Wie die Pflegeheimbewohner*innen den ersten Lockdown und Sommer 2020 erlebten, wie sehr sie durch Sorgen und Ängste belastet waren, und welche Rolle soziale Kontakte beim Umgang mit diesen Sorgen spielten, ist bisher kaum quantitativ erforscht worden. Hier setzt der vorliegende Beitrag an und präsentiert die Ergebnisse einer repräsentativen Befragung von 259 Pflegeheimbewohner*innen in Österreich, um die folgende Forschungsfrage zu beantworten: Wie beeinflussten die sozialen Beziehungen von Pflegeheimbewohner*innen ihre Corona-Sorgen während des ersten Lockdowns und des Sommers 2020? Dabei wird folgende Hypothese überprüft: Pflegeheimbewohner*innen, die einen weniger engen Kontakt zu anderen Personen (Angehörigen, Pflegekräften, andere Bewohner*innen) und eine stärkere Einsamkeit während der Pandemie angeben, weisen eher Corona-Sorgen auf.

## Methodisches Vorgehen

Es wurde eine repräsentative Face-to-face-Befragung mit Bewohner*innen stationärer Pflegeeinrichtungen eines Pflegeheimträgers in Österreich durchgeführt. Die Stichprobenziehung erfolgte mithilfe der Probability-Proportional-to-Size(PPS)-Methode [11], wodurch zufällig 16 Häuser der insgesamt 62 Einrichtungen ausgewählt wurden. In jedem der 16 Häuser erstellten die Pflegekräfte eine Gesamtliste aller Bewohner*innen, aus dieser wurden anschließend zufällig Bewohner*innen für die Befragung gezogen. Die Bewohner*innen mussten folgenden Kriterien erfüllen: Bewohner*innen befanden sich zum Erhebungszeitpunkt seit mindestens 3 Monaten in Langzeitpflege, hatten keine Erwachsenenvertretung und wiesen eine gute Kommunikationsfähigkeit auf. Um auch Personen mit leichter bis mittlerer Demenzerkrankungen zu berücksichtigen, wurde ein Wert von mindestens 17 im Mini-Mental-Status-Test (MMST) als Einschlusskriterium festgelegt. Der MMST wird von den Pflegekräften bei Verdacht auf eine demenzielle Erkrankung durchgeführt. Es konnten eine Ausschöpfungsquote von 79 % erreicht und insgesamt 259 Bewohner*innen im Zeitraum von August bis Mitte September 2020 befragt werden (Zusammensetzung der Stichprobe: Tab. 1).

**Table 1:** 

	Anzahl *(n*)	Anteil (%)
**Geschlecht**		
Männlich	67	26
Weiblich	192	74
**Alter** (Jahre)		
Bis 69	23	9
70–79	59	23
80–89	111	43
Ab 90	66	25
**Bildungsabschluss**		
Bis Pflichtschulabschluss (ISCED-11: 0–2)	136	53
Mittlere Bildung ohne Matura (ISCED-11: 3)	86	34
Höhere Bildung ab Matura (ISCED-11: 4–8)	34	13
(Fehlend)	3	
**Benötigte Unterstützung bei Aktivitäten des täglichen Lebens (ADL)**		
Selbstständig bei allen Tätigkeiten (0)	54	21
1	59	23
2	52	20
3	78	30
Bei allen Tätigkeiten wird Hilfe benötigt (4)	13	5
(Fehlend)	3	

*ISCED* International Standard Classification of Education

Der Fragebogen ist maßgeblich an die SHARE(Survey of Health, Ageing and Retirement in Europe)-Erhebung zu COVID-19 [22] und die Erhebung der Lebensqualität in Pflegeheimen [3] angelehnt. Bei der Erstellung wurden zentrale Kriterien in Bezug auf die Besonderheit der Zielgruppe beachtet. Die Interviews fanden face-to-face statt, geachtet wurde auf einfache Formulierungen der Fragen, ein geringeres Differenzierungsniveau der Antworten und eine kurze Durchführungsdauer [1]. Dementsprechend wurden die meisten verwendeten Variablen auf Ja/Nein-Antworten reduziert und die Skalen auf maximal 3 Antwortmöglichkeiten vereinfacht. Zur Validierung des standardisierten Fragebogens wurden kognitive Pretests mit 5 Pflegeheimbewohner*innen durchgeführt und darauf aufbauend Showcards mit den Antwortkategorien in großer Schrift gedruckt und eine Zeitachse erstellt, um den Zeithorizont der jeweiligen Frage (vor Corona, erster Lockdown oder Sommer 2020) sichtbar darzustellen.

Corona-Sorgen wurden in Anlehnung an das deutsche modifizierte Health Anxiety Inventory [4] erfragt, wobei in der vorliegenden Analyse das Item „Ich mache mir viele Sorgen über das Coronavirus“ (Nein: 0; Ja: 1) als Operationalisierung der Corona-Sorge herangezogen wurde. Soziale Beziehungen wurden mithilfe mehrerer Variablen abgefragt. Die Beziehung zu den Angehörigen wurde über die Häufigkeit des persönlichen und telefonischen Kontakts (2: oft; 1: manchmal; 0: nie) getrennt voneinander abgefragt, und anschließend wurde durch Addition ein metrischer Summenindex von 0: nie bis 4: oft erstellt. Für die Messung der Beziehung zu den Mitbewohner*innen und Pflegekräften wurden in Anlehnung an Amann et al. [3] die Fragen gestellt: „Haben Sie in der Phase der strengen Schutzmaßnahmen mit anderen Bewohner*innen/dem Personal über Ihre Sorgen und Ängste gesprochen?“ (Nein: 0; Ja: 1). Schließlich wurden Fragen zur erlebten Einsamkeit gestellt, wobei zwischen sozialer und emotionaler Einsamkeit unterschieden wurde. Für die Operationalisierung wurde die Skala von De Jong Gierveld und Van Tilburg [10] gekürzt, und die Antwortmöglichkeiten wurden vereinfacht. Im Folgenden werden das Item „Ich habe Leute vermisst, bei denen ich mich wohl fühle (Nein: 0; Ja: 1)“ als Maß der emotionalen Einsamkeit und das Item „Es gab immer jemanden in meiner Umgebung, mit dem ich die Situation besprechen konnte (Ja: 0; Nein: 1)“ als Maß der sozialen Einsamkeit angeführt.

Die Daten wurden mithilfe von IBM SPSS Statistics Version 27 (IBM, Armonk, NY, USA) ausgewertet. Zur bivariaten Analyse wurden je nach Skalenniveau entweder *t*- oder χ^2^-Tests angewendet. Aufgrund der dichotomen, abhängigen Variable wurde eine binär-logistische Regression durchgeführt; diese weist eine akzeptable Modellgüte auf (*n* = 231; Nagelkerkes‑R^2^ = 0,146, Hosmer-Lemeshow-Test [χ^2^ = 5,02; df = 8; *p* = 0,756] und 65 % richtig klassifizierte Fälle im Einschlussmodell im Vergleich zu 60 % im Anfangsmodell).

## Ergebnisse

Die Mehrheit der befragten Bewohner*innen hatte im Sommer 2020 engen Kontakt zu ihren Angehörigen. So hatten 61 % der Bewohner*innen oft persönlichen und/oder telefonischen Kontakt zu Angehörigen, und weitere 22 % gaben an, manchmal Kontakt zu haben (Tab. 2). Die anderen Bewohner*innen und das Personal spielten jedoch nur für ein knappes Drittel (28 % bzw. 33 %) eine Rolle im Umgang mit Ängsten und Sorgen. Des Weiteren fühlten sich 74 % der Bewohner*innen sozial eingebunden und teilten mit, dass es immer jemanden in ihrer Umgebung gab, mit dem sie über die Situation sprechen konnten.

**Table 2:** 

	Anzahl (*n*)	Anteil (%)
**Corona-Sorge**		
Nein	111	43
Ja	145	57
(Fehlend)	3	
Über Ängste und Sorgen gesprochen (mit Bewohner*innen)		
Nein	178	72
Ja	70	28
(Fehlend)	11	
**Über Ängste und Sorgen gesprochen (mit Personal)**		
Nein	173	67
Ja	85	33
(Fehlend)	1	
**Kontakthäufigkeit mit Angehörigen (telefonisch oder persönlich)**		
Nie (0)	20	8
1	23	9
2	56	22
3	61	24
Oft (4)	98	38
(Fehlend)	1	
**Emotionale Einsamkeit: „Ich habe Leute vermisst, bei denen ich mich wohl fühle“**		
Nein	101	39
Ja	156	61
(Fehlend)	2	
**Soziale Einsamkeit: „Es gab immer jemanden in meiner Umgebung, mit dem ich die Situation besprechen konnte“**		
Nein	66	26
Ja	185	74
(Fehlend)	8	

Die Pandemie stellte v. a. eine emotionale Belastung dar. So gaben 57 % der Teilnehmenden an, dass sie sich allgemein Sorgen über das Virus machen, und 61 % fühlten sich emotional einsam. Gleichzeitig bedeutet dies allerdings auch, dass 43 % der befragten Pflegeheimbewohner*innen sich keine Sorgen über COVID-19 machen und 39 % nicht emotional einsam sind.

Bei der Überprüfung des Einflusses des Kontakts zu Personen außerhalb der Pflegeeinrichtung ergibt sich bivariat – im Gegensatz zur formulierten Hypothese – ein signifikanter, positiver Effekt. Personen, die sich allgemein Sorgen über das Virus machten, gaben mit einem Mittelwert (MW) von 2,50 (Standardabweichung [SD] ± 0,59) einen signifikant häufigeren Kontakt zu Angehörigen an als Personen, die keine Sorgen angegeben hatten (MW = 2,23, SD ±0,66; *p* < 0,001). Aufgrund des Querschnittsdesigns der Stichprobe kann jedoch die Richtung des Zusammenhangs nicht überprüft werden: So machen sich entweder Personen mit mehr Kontakt auch mehr Sorgen, oder Personen mit größeren Sorgen suchen mehr Kontakt.

In Bezug auf die Einsamkeit konnte die formulierte Hypothese bestätigt werden. Es besteht ein bivariater, positiver Zusammenhang zwischen der Corona-Sorge und der emotionalen Einsamkeit (φ = 0,255; *p* < 0,001). Bewohner*innen, die Menschen vermisst hatten, bei denen sie sich wohl fühlen, gaben auch häufiger Sorgen über die Corona-Pandemie an. Auch hier kann allerdings keine Überprüfung der Richtung des Zusammenhangs erfolgen. Im Gegensatz zur emotionalen Einsamkeit besteht jedoch kein Zusammenhang zwischen der Corona-Sorge und der sozialen Einsamkeit (φ = 0,058; *p* > 0,05). Zudem konnte auch kein signifikanter Zusammenhang zwischen der Corona-Sorge und dem Sprechen über Sorgen und Ängste mit anderen Bewohner*innen und Pflegekräften gefunden werden.

Schließlich wurde ein binär-logistisches Regressionsmodell zur Vorhersage von Corona-Sorgen berechnet (Tab. 3). Unter Einbezug mehrerer Variablen zeigt sich, dass die emotionale Einsamkeit einen positiven Effekt auf die Corona-Sorge hat, emotionale Einsamkeit und höhere Corona-Sorgen also signifikant zusammenhängen („odds ratio“ [OR] = 2,30; Konfidenzintervall [95 %-KI] = 1,26–4,25). Gleichzeitig machen sich Personen mit viel Kontakt zu ihren Angehörigen sich auch mehr Sorgen (OR = 1,32; 95 %-KI = 1,04–1,67).

**Table 3:** 

	Exp(B)	95 %-KI	*p*
(Regressionskonstante)	0,15	0,04–0,56	0,005
Mit Bewohner*innen über Ängste und Sorgen gesprochen (Ref.: Nein)	1,02	0,51–2,06	0,954
Mit Personal über Ängste und Sorgen gesprochen (Ref.: Nein)	1,22	0,64–2,35	0,539
Kontakthäufigkeit mit Angehörigen (0: nie bis 4: oft)	**1,32**	**1,04–1,67**	**0,022**
Emotionale Einsamkeit: „Ich habe Leute vermisst, bei denen ich mich wohl fühle“ (Ref.: Nein)	**2,30**	**1,26–4,25**	**0,007**
Soziale Einsamkeit: „Es gab immer jemanden in meiner Umgebung, mit dem ich die Situation besprechen konnte“ (Ref.: Nein)	1,06	0,53–2,16	0,865
**Geschlecht (Ref. männlich)**			
Weiblich	1,07	0,53–2,13	0,854
**Alter (Jahre; Ref. ab 90 Jahre)**			
Bis 79	1,05	0,49–2,26	0,899
80–89	1,92	0,95–3,95	0,072
**Bildung (Ref. Matura oder höher (ISCED-11: 4–8))**			
Bis Pflichtschulabschluss (ISCED-11: 0–2)	1,75	0,72–4,29	0,219
Mittlere Bildung ohne Matura (ISCED-11: 3)	1,55	0,63–3,83	0,341
Aktivitäten des täglichen Lebens (ADL; 0: selbstständig bis 4: Hilfe bei allen Tätigkeiten)	1,12	0,88–1,43	0,356

*n* = 231, Nagelkerkes Pseudo‑R^2^ = 0,15, fett-geschriebene Zahlen sind signifikant

*Exp(B)* ist das Exponential des Regressionskoeffizienten und gibt die „Odds Ratios“ an, *ISCED* International Standard Classification of Education, *95* *%-KI* 95 %-Konfidenzintervall

## Diskussion

In diesem Beitrag wurde der Einfluss der sozialen Beziehungen auf die Corona-Sorge bei Bewohner*innen von stationären Pflegeeinrichtungen untersucht. Die präsentierten Daten verdeutlichen, dass die Lebenswelten der befragten Pflegeheimbewohner*innen im ersten Lockdown und im Sommer 2020 von emotionaler Einsamkeit und Sorgen geprägt waren, denn die Mehrheit der Befragten gibt an, sich Sorgen über das Virus zu machen und sich emotional einsam zu fühlen. Die Daten entsprechen anderen Ergebnissen qualitativer Forschung über die Auswirkungen der Pandemie auf Bewohner*innen von stationären Pflegeeinrichtungen [5, 14, 19]. Allerdings gab in der vorliegenden Studie auch eine recht große Gruppe der Befragten an, nicht sozial oder emotional einsam zu sein (74 und 39 %) und sich auch keine Sorgen über COVID-19 zu machen (43 %). Dies spricht für eine psychische Resilienz einiger Pflegeheimbewohner*innen und weist darauf hin, dass nicht nur ältere Menschen im Privathaushalt besser mit den psychologischen Belastungen der Pandemie umgehen können als ursprünglich erwartet [6, 15], sondern auch ein großer Teil der Pflegeheimbewohner*innen. Diese Resilienz bei Pflegeheimbewohner*innen, die die Daten andeuten, sollte in weiterführenden Studien näher beleuchtet werden. Sie könnte einerseits an der umfangreichen Lebenserfahrung im Umgang mit Krisen und Problemen liegen [2], andererseits auch am geschützten Raum des Pflegeheims, der sowohl soziale Einbindung als auch Sicherheit während des ersten Lockdowns bieten konnte [16].

In Bezug auf den Einfluss der sozialen Beziehungen auf die Corona-Sorge lassen sich zwei Ergebnisse festhalten: Es besteht ein positiver Zusammenhang zwischen der emotionalen Einsamkeit und der Corona-Sorge, jedoch nicht zwischen der sozialen Einsamkeit und der Corona-Sorge. Lediglich die emotionale Verbundenheit mit dem sozialen Umfeld hat eine protektive Wirkung gegenüber Corona-Sorgen, weshalb davon ausgegangen werden kann, dass die Besuchsverbote für Angehörige sich v. a. auf die emotionale Einsamkeit ausgewirkt haben. In der Langzeitpflege wurde die schwer zu erreichende Kompensation von fehlender, emotionaler Nähe durch Pflegekräfte bereits vor der Pandemie thematisiert [20]. Die Pandemie hat die Thematik jedoch verschärft und aufgezeigt, dass es neue Konzepte der psychischen Unterstützung im Pflegeheim braucht, die v. a. den Aufbau von emotionaler Nähe zwischen Bewohner*innen und Pflegekräften fördern.

Des Weiteren zeigen die Daten, dass ein intensiver telefonischer und/oder persönlicher Kontakt zu Angehörigen ebenfalls mit höheren Corona-Sorgen zusammenhängt. Für diesen Zusammenhang könnte es unterschiedliche Erklärungen geben, die mit der vorliegenden Studie nicht eindeutig ausgeschossen werden können. In der Literatur lassen sich ähnliche Ergebnisse in der Allgemeinpopulation finden: So konnte gezeigt werden, dass der Kontakt mit Personen außerhalb des Haushalts mit verstärkter psychologischer Belastung während der Pandemie zusammenhängt [17]. Eine weitere Studie fand, dass die Sorge um das Wohlergehen von nahestehenden Personen außerhalb des eigenen Haushalts die häufigste während der Pandemie war und wesentlich weniger häufig die Angst vor einer eigenen Infektion bestand [24]. Neben den Sorgen um andere Personen könnte auch der umgekehrte Fall zutreffen, nämlich dass Personen, die sich Sorgen machen, sich auch eher an ihre Angehörigen wenden. Es löst also nicht der Kontakt höhere Sorgen aus, sondern Personen, die sich mehr Sorgen machen, suchen öfter Kontakt, um mit diesen Sorgen umgehen zu können. In einer Studie mit älteren Menschen im Privathaushalt nennen die Befragten das Suchen nach Kontakt mit Angehörigen und Freund*innen als eine der wichtigsten Coping-Strategien im Umgang mit den pandemiebedingten Belastungen [13]. Dies entspricht auch der sozioemotionalen Selektionstheorie [7], wobei der Kontakt zu nahestehenden Personen zur Regulation von Sorgen und Ängsten beiträgt. Während andere Bewohner*innen und Pflegekräfte kaum eine Rolle für die pandemiebedingten emotionalen Belastungen spielen, muss die Rolle der Angehörigen noch stärker untersucht werden. Mehr Wissen darüber, ob die Angehörigen einen Ausgangspunkt für Sorgen oder eine Ressource für einen besseren Umgang mit psychischen Belastungen darstellen, ist in den aktuellen Zeiten, in denen eine Krise die nächste ablöst, für die Planung von Interventionen wichtig.

## Limitationen

Quantitative Befragungen im Pflegeheim müssen der Zielgruppe angepasst werden und unterliegen gewissen Limitationen. Der Fragebogen musste speziell entwickelt werden, wobei v. a. die Reduzierung der Skalen auf 2 bis 3 Antwortmöglichkeiten die Auswertung der Ergebnisse einschränkte, da nur bestimmte Auswertungen ausgeführt werden konnten. Für die Interpretation der Ergebnisse bedeutet die geringere Varianz der Antworten auch, dass die Heterogenität der Erfahrungen nur beschränkt erfasst werden konnte. Außerdem musste eine Vorauswahl der Bewohner*innen getroffen werden, da für die Teilnahme gewisse kognitive und kommunikative Kompetenzen erforderlich waren. Diese Vorauswahl bedingte jedoch den Ausschluss von Bewohner*innen, deren Erfahrungen nun nicht in den Ergebnissen repräsentiert sind. Dies betrifft v. a. Menschen mit einer Demenzerkrankung und starken Hör- oder Sprechproblemen. Des Weiteren repräsentieren die Ergebnisse lediglich Bewohner*innen eines Pflegeheimträgers in Österreich und nicht die Grundgesamtheit aller Pflegeheimbewohner*innen. Da die Träger oft unterschiedliche Strategien zum Umgang mit COVID-19 umgesetzt haben, können die Erfahrungen in verschiedenen Pflegeheimen unterschiedlich ausfallen. Schließlich ist als Limitation das Querschnittsdesign der Studie zu nennen, das eine Überprüfung von Kausalitäten nicht zulässt.

## Fazit für die Praxis

- Die Mehrheit der befragten Pflegeheimbewohner*innen gibt an, sich emotional einsam zu fühlen und sich Sorgen über die „coronavirus disease 2019“ (COVID-19) zu machen. Den meisten Befragten ist es allerdings gelungen, den Kontakt zu ihrem sozialen Umfeld aufrechtzuerhalten und somit eine soziale Einsamkeit zu verhindern.
- Emotionale Einsamkeit hängt unter Pflegeheimbewohner*innen mit höheren Corona-Sorgen zusammen, während soziale Einsamkeit keinen signifikanten Einfluss auf die Corona-Sorgen hat.
- Der Einfluss des Kontaktes zu Angehörigen ist nicht eindeutig zu interpretieren; er könnte sowohl ein Ausgangspunkt für Sorgen und Ängste in Bezug auf die Pandemie sein als auch als Coping-Strategie für den Umgang mit Sorgen dienen.
